# Serum Anti-Collagen IV IgM and IgG Antibodies as Indicators of Low Vascular Turnover of Collagen IV in Patients with Long-Term Complications of Type 2 Diabetes

**DOI:** 10.3390/diagnostics11050900

**Published:** 2021-05-19

**Authors:** Krasimir Kostov, Alexander Blazhev

**Affiliations:** 1 Department of Pathophysiology, Medical University-Pleven, 1 Kliment Ohridski Str., 5800 Pleven, Bulgaria; 2 Department of Biology, Medical University-Pleven, 1 Kliment Ohridski Str., 5800 Pleven, Bulgaria; yalishanda9@gmail.com

**Keywords:** type 2 diabetes, collagen IV, vascular basement membrane, matrix metalloproteinases-2 and -9, diabetic vascular complications

## Abstract

Thickening of the vascular basement membrane (BM) is a fundamental structural change in the small blood vessels in diabetes. Collagen type IV (CIV) is a major component of the BMs, and monitoring the turnover of this protein in type 2 diabetes (T2D) can provide important information about the mechanisms of vascular damage. The aim of the study was through the use of non-invasive biomarkers of CIV (autoantibodies, derivative peptides, and immune complexes) to investigate vascular turnover of CIV in patients with long-term complications of T2D. We measured serum levels of these biomarkers in 59 T2D patients with micro- and/or macrovascular complications and 20 healthy controls using an ELISA. Matrix metalloproteinases-2 and -9 (MMP-2 and MMP-9) were also tested. In the T2D group, significantly lower levels of CIV markers and significantly higher levels of MMP-2 and MMP-9 were found compared to controls. A significant positive correlation was found between IgM antibody levels against CIV and MMP-2. These findings suggest that vascular metabolism of CIV is decreased in T2D with long-term complications and show that a positive linear relationship exists between MMP-2 levels and CIV turnover in the vascular wall.

## 1. Introduction

Diabetes mellitus is a chronic disease with an increasing frequency in recent decade. The International Diabetes Federation (IDF) estimates that the global number of people with diabetes will increase to 693 million by 2045 [1]. Type 2 diabetes (T2D) accounts more than 90% of all diagnosed diabetes cases and is among the leading causes of cardiovascular morbidity and mortality [2]. In patients with T2D, the treatment of cardiovascular disease can be improved by identifying specific biomarkers to assess vascular changes [3].

Collagen (COL) is one of the primary load bearing components in the arterial wall and plays an important role in vascular function in both normal and pathological processes [4,5]. In patients with T2D, monitoring the turnover of this structural protein may provide important information about the mechanisms of vascular damage [6,7,8,9,10,11,12]. As a result of degradation processes occurring in the vascular extracellular matrix (ECM), the released COL peptides enter the circulation and can be detected and examined in the serum [13]. In this regard, the measurement of non-invasive markers of COL degradation, such as specific autoantibodies (autoAbs), COL-derived peptides (DP), and circulating immune complexes (CIC) of the COL, may be useful for monitoring the development of vascular complications in T2D [14,15,16].

Basement membranes (BMs) are a main focus of scientific research due to their role in the pathogenesis of a number of diseases [17]. Diabetes is a “BM disease”, in which microvascular damage of the capillaries is characterized by thickening of the BMs [18,19,20]. COL type IV (CIV) is a major vascular BM protein and represents up to 50% of all BM proteins [21,22]. Unlike fibrillar COLs (I, II, and III), CIV forms a network structure and is considered to be crucial for vascular BM assembly and stability [23]. There are six α chains (α1–α6) that can be used to make the trimers of CIV. Predominant isoform, however, consists of two α1 and one α2 chain [α1(IV)]_2_α2(IV) [24,25,26]. Inside cells, the three α-chains assemble forming triple helical molecules, termed protomers, which oligomerize outside the cells into a supramolecular network [27].

An important factor for the development of vascular complications in T2D is the imbalanced turnover of CIV in the vessel wall [8,10,11,12,14]. As a result of the underlying disease, CIV can acquire immunogenic properties and the ability to induce a humoral immune response [13,28,29]. Processing of CIV by matrix metalloproteinases (MMPs) gives rise to the release of fragments that are able to behave as epitopes, since they can be bound by circulating Abs, forming CIC of CIV (CIC-CIV) [30,31]. Proteolysis of CIV is accompanied by the release of DP (CIV-DP) into the bloodstream, which is followed by the production of specific anti-CIVAbs (ACIVAbs) from IgM, IgG, and IgA classes (ACIVAbs IgM, ACIVAbs IgG, and ACIVAbs IgA) [14,32,33]. These autoAbs can serve as valuable control biomarkers for the turnover of CIV in the vascular ECM. The coexistence of T2D and hypertension (HTN) further disrupts the metabolism of CIV, leading to greater stiffness of the small arteries and enhanced structural alterations in the systemic microcirculation [12].

To examine CIV turnover in the vascular ECM, we tested the serum levels of ACIVAbs IgM, ACIVAbs IgG, CIV-DP, CIC-CIV, free ACIVAbs, MMP-2, and MMP-9 in patients with T2D by ELISA, comparing the results with those of matched healthy control group.

## 2. Materials and Methods

### 2.1. Characteristics of the Study Population

The study population consisted of 79 people: Control group (*n* = 20; mean age 61.5 ± 11.4 years), individuals without diabetes mellitus, HTN, or vascular diseases; T2D group (*n* = 59; mean age 60.8 ± 14.7 years; mean disease duration of 10.1 ± 7.8 years), patients with long-term micro- and/or macrovascular complications. The number of patients was higher than that of controls to ensure greater statistical representation of the population with T2D vascular complications. The incidence of microangiopathy in the T2D group (*n* = 50) was 85%, and the incidence of macroangiopathy (*n* = 18) was 31%. Nine patients had both microvascular and macrovascular disease. The clinical characteristics of the groups are shown in Table 1.

### 2.2. Screening of the Patients and Controls

The patients and control participants were screened for microangiopathy using ophthalmoscopy and/or assessment of 24 h urine albumin excretion. Macroangiopathy in the T2D group was evaluated on the basis of clinical evidence for coronary artery disease, cerebrovascular disease, peripheral arterial disease, and/or history for acute arterial vascular events. Control persons were screened for macroangiopathy by physical examination, blood pressure (BP) measurement, electrocardiogram (ECG) testing, measuring cholesterol levels, data on obesity and smoking, and family history.

### 2.3. Immunological and Biochemical Assays

To measure the levels of ACIVAbs, CIV-DP, CIC-CIV, free ACIVAbs, MMP-2, MMP-9, and the other laboratory parameters, blood was drawn into tubes containing a clot activator and was centrifuged at 2500 rpm for 10 min to separate the serum. Until the immunological assay, the serums were stored at −70 °C.

#### 2.3.1. Determination of ACIVAbs IgM and ACIVAbs IgG

To measure the ACIVAbs IgM and ACIVAbs IgG concentrations, a sandwich ELISA was used. The assays were performed as follows: microtiter 96-well polystyrene plates were coated with 100 μL of 10 μg/mL of human CIV (Sigma-Aldrich, St. Louis, MO, USA), followed by an overnight incubation at 4 °C. Then, 100 μL serum sample (diluted 1:10) was placed in each well of the microtiter plates and incubated for 1 h at 37 °C. After washing, 100 μL of goat anti-human IgM Ab, Fc5µ, HRP conjugate (AP114P, Sigma-Aldrich, St. Louis, MO, USA), or goat anti-human IgG Ab, Fc, and HRP conjugate (AP113P, Sigma-Aldrich, St. Louis, MO, USA), respectively, were added to each well for 1 h at 37 °C. All immunoconjugates were diluted 1:10,000. Then, 100 μL of ortho-phenylenediamine (4 mg/mL in 0.05 M citrate buffer) was added as a colorimetric substrate for 30 min. The reaction was stopped by adding 50 μL of 4 M H_2_SO_4_ to each well, and the optical density was measured with a micro-ELISA plate reader (Coulter Microplate Reader UV Max, Molecular Devices Corp., Menlo Park, CA, USA) at a wavelength of 492 nm.

#### 2.3.2. Determination of CIV-DP

To measure CIV-DP concentrations, a sandwich ELISA was used. The assay was performed as follows: each well of the microtiter plate was sensitized with 100 μL of 10 μg/mL of mouse monoclonal antibody to collagen IV [COL-94] (Cat. No. ab6311, Abcam, Cambridge, UK), followed by an overnight incubation at 4 °C. Then, 100 μL serum sample (diluted 1:5) was placed in each well of a microtiter plate and incubated for 1 h at 37 °C. After washing, 100 μL of rabbit anti-human CIV polyclonal antibody (Cat. No. ab6586, Abcam, Cambridge, UK) (diluted 1:2000) was allowed to react in each well at 37 °C for 1 h. After washing, peroxidase-conjugated goat anti-rabbit IgG H&L (HRP) (Cat. No. ab205718, Abcam, Cambridge, UK) diluted 10,000 fold was added to each well. The plate was incubated for 1 h at 37 °C. Then, 100 μL of ortho-phenylenediamine (4 mg/mL in 0.05 M citrate buffer) was added as a colorimetric substrate for 30 min. The reaction was stopped by adding 50 μL of 4 M H_2_SO_4_ to each well, and the optical density was measured with a micro-ELISA plate reader (Coulter Microplate Reader UV Max, Molecular Devices Corp., Menlo Park, CA, USA) at a wavelength of 492 nm.

#### 2.3.3. Determination of CIC-CIV

For the determination of CIC-CIV, a method based on C3-binding glycoprotein was used: Complement-Inhibiting Factor (CIF)-Enzyme Linked Immunosorbent Assay (CIF-ELISA) [16]. The assay was performed as follows: microtiter 96-well polystyrene CIF-ELISA plates were prepared by incubation of the wells with CIF (20 μg/mL in 0.2 M carbonate-bicarbonate buffer, pH 9.6) overnight at 4 °C. After repeatedly washed in order to remove unbound CIF, human sera (100 μL) were added to the plates and incubated for 1 h at 37 °C. After washing, 100 μL of rabbit anti-human CIV polyclonal antibody (Cat. No. ab6586, Abcam, Cambridge, UK) (diluted 1:2000) was allowed to react in each well at 37 °C for 1 h. After washing, peroxidase-conjugated goat anti-rabbit IgG H&L (HRP) (Cat. No. ab205718, Abcam, Cambridge, UK) diluted 10,000 fold was added to each well. The plate was incubated for 1 h at 37 °C. Then, 100 μL of ortho-phenylenediamine (4 mg/mL in 0.05 M citrate buffer) was added as a colorimetric substrate for 30 min. The reaction was stopped by adding 50 μL of 4 M H_2_SO_4_ to each well, and the optical density was measured with a micro-ELISA plate reader (Coulter Microplate Reader UV Max, Molecular Devices Corp., Menlo Park, CA, USA) at a wavelength of 492 nm.

#### 2.3.4. Determination of Free ACIVAbs

Circulating free ACIVAbs were determined using a two-step method [34]: (1) CIF-ELISA for elimination of the immune complexes of collagen IV, followed by (2) Collagen IV-specific ELISA for determination of free anti-collagen IV Ab. The assay was performed as follows: microtiter 96-well polystyrene CIF-ELISA plates were prepared by incubation of the wells with CIF (20 μg/mL in 0.2 M carbonate-bicarbonate buffer, pH 9.6) overnight at 4 °C. After repeatedly washed in order to remove unbound CIF, human sera (100 μL) were added to the plates and incubated for 1 h at 37 °C. At the end of the incubation period, the samples were transferred from the CIF plates to the 96-well polystyrene plate coated with 100 μL of 10 μg/mL of human CIV (#CC076 Sigma-Aldrich, St. Louis, MO, USA) at room temperature for 3 h. After washing, 100 μL of rabbit monoclonal [H169-1-5] anti-human IgG Fc (Cat. No. ab125909, Abcam, Cambridge, UK) (diluted 1:2000) were added to each well for 1 h at 37 °C. The wells were washed and peroxidase-conjugated goat anti-rabbit IgG H&L (HRP) (Cat. No. ab205718, Abcam, Cambridge, UK) diluted 10,000 fold was then added to each well. Then, 100 μL of ortho-phenylenediamine (4 mg/mL in 0.05 M citrate buffer) was added as a colorimetric substrate for 30 min. The reaction was stopped by adding 50 μL of 4 M H_2_SO_4_ to each well, and the optical density was measured with a micro-ELISA plate reader (Coulter Microplate Reader UV Max, Molecular Devices Corp., Menlo Park, CA, USA) at a wavelength of 492 nm.

#### 2.3.5. Determination of MMP-2 and MMP-9

To measure MMP-2 and MMP-9 concentrations, ELISA kits from R&D Systems (Cat. No. DMP2F0 and Cat. No. DMP900) (Minneapolis, MN, USA) were used according to the manufacturer’s instructions. Serum samples were assayed at 450 nm on an automatic micro-ELISA plate reader (Coulter Microplate Reader UV Max, Molecular Devices Corp., Menlo Park, CA, USA).

#### 2.3.6. Biochemical Analysis

The assays were performed using automatic biochemistry analyzer. HbA1c and CRP were measured by a turbidimetric immunoassay. Enzymatic methods were used to measure of total cholesterol (TC), low-density lipoprotein cholesterol (LDL-C), high-density lipoprotein cholesterol (HDL-C), and triglyceride (TG).

### 2.4. Clinical Tests and Procedures

All patients were subjected to routine nephrologic (renal ultrasound, creatinine, blood urea nitrogen, urinary albumin excretion), ophthalmic (visual acuity test, ophthalmoscopy, tonometry), and neurologic (muscle reflexes, electromyography) examination. BP was measured using a standard cuff mercury sphygmomanometer on the left arm in a sitting position, after a 5–10 min rest. Normal BP was defined as SBP 120–129 mmHg and DBP 80–84 mmHg. HTN was defined as SBP ≥ 140 mmHg and/or DBP ≥ 90 mmHg. A twelve-lead ECG was performed using standard equipment. Body mass index (BMI) was calculated, using the standard metric BMI formula (Kg/m^2^). BMI between 18.5–24.9 was considered normal, 25–29.9 was considered overweight, and ≥30 was considered obese.

### 2.5. Statistical Analysis

Statistical analyses were performed using SPSS 23.0 software (SPSS, Inc., Chicago, IL, USA). The data were expressed as mean ± standard deviation (SD). The differences between the groups were assessed by Student’s unpaired *t*-test. Correlation analysis was performed with Pearson’s correlation test. Values of *p* < 0.05 were considered statistically significant.

## 3. Results

### 3.1. Comparison of the Levels of ACIVAbs IgM and ACIVAbs IgG between the T2D and Control Groups

**Figure 1:**
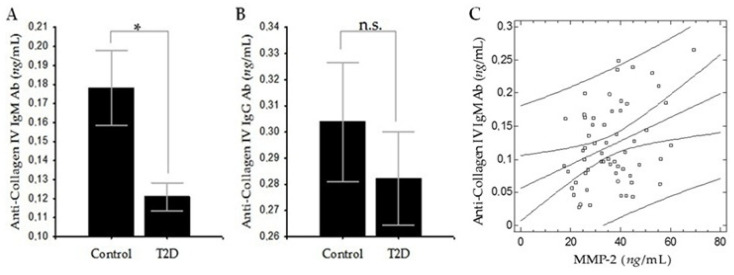
(**A**) Serum levels of ACIVAbs IgM in T2D group vs. control group. (**B**) Serum levels of ACIVAbs IgG in T2D group vs. control group. (**C**) Relationship between ACIVAbs IgM and MMP-2 in the T2D group. Data are represented as mean ± SD. * *p* < 0.05, n.s.—not significant.

To measure serum levels of ACIVAbs IgM and ACIVAbs IgG by ELISA, goat anti-human IgM Ab, Fc5 µ, horseradish peroxidase (HRP) conjugate or goat anti-human IgG Ab, Fc, and HRP conjugate, respectively, were used. The levels of ACIVAbs IgM were significantly lower in the T2D group compared to healthy controls (0.12 ± 0.06 vs. 0.18 ± 0.09; *p* = 0.016; Figure 1A). The levels of ACIVAbs IgG were also lower in the T2D group than in controls, but the difference was not statistically significant (0.28 ± 0.14 vs. 0.30 ± 0.11; *p* = 0.54; Figure 1B). We also found a positive correlation between ACIVAbs IgM and MMP-2 in the T2D group (*r* = 0.343; *p* = 0.008; Figure 1C).

### 3.2. Comparison of the Levels of CIV-DP, CIC-CIV, and Free ACIVAbs IgG between the T2D and Control Groups

**Figure 2:**
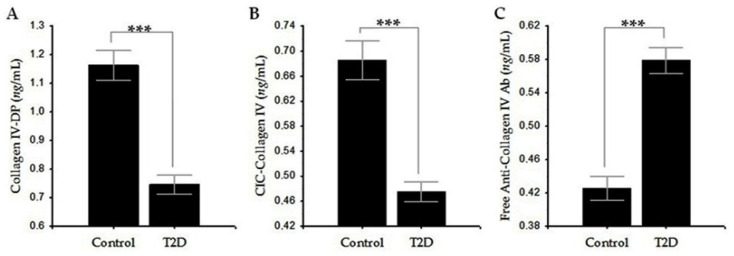
(**A**) Serum levels of CIV-DP in T2D group vs. control group. (**B**) Serum levels of CIC-CIV in T2D group vs. control group. (**C**) Serum levels of free ACIVAbs IgG in T2D group vs. control group. Data are represented as mean ± SD. *** *p* < 0.001.

To measure serum levels of CIV-DP by ELISA, rabbit anti-human CIV polyclonal Ab and peroxidase-conjugated goat anti-rabbit IgG were used. We found that CIV-DP levels were significantly lower in T2D group compared to healthy controls (0.74 ± 0.27 vs. 1.16 ± 0.25; *p* < 0.001; Figure 2A). To measure CIC-CIV levels, a method based on C3-binding glycoprotein was used: Complement-Inhibiting Factor (CIF)-Enzyme Linked Immunosorbent Assay (CIF-ELISA) [16]. CIF catches out different CIC in the serum, and to differentiate only CIC-CIV, we used rabbit anti-human CIV polyclonal IgG Abs. We found that CIC-CIV levels were also significantly reduced in T2D group than in controls (0.47 ± 0.13 vs. 0.68 ± 0.15; *p* < 0.001; Figure 2B). Circulating free ACIVAbs IgG were determined using a two-step method [34]: 1) CIF-ELISA for elimination of CIC-CIV, followed by 2) CIV-specific ELISA for determination of free ACIVAbs IgG, using rabbit monoclonal anti-human IgG, Fc. The results show that the levels of free ACIVAbs IgG in the T2D group were significantly higher compared to those of the control group (0.58 ± 0.13 vs. 0.42 ± 0.07; *p* < 0.001; Figure 2C). It should be noted that ACIVAbs IgM and ACIVAbs IgG as biomarkers reflect common levels of these classes of ACIVAbs measured in controls and patients with T2D. The difference between ACIVAbs IgG and free ACIVAbs IgG as biomarkers is that the latter does not reflect the total levels of ACIVAbs IgG, but only that part of ACIVAbs IgG that does not participate in the formation of CIC-CIV. 

## 4. Discussion

Vascular BM thickening is a fundamental structural alteration of small blood vessels in diabetes. Research has established hyperglycemia as the primary causal factor mediating this alteration [35]. BM thickening is most evident in the eye (diabetic retinopathy, DR) [18,35] and the kidney (diabetic nephropathy, DN) [20,36]. Changes in the BMs of retinal vessels in DR may develop along with or without DN [36]. It is assumed that excess synthesis and/or decreased degradation of BM components are a major contributing factors to its thickening [35,37,38,39]. Metabolic dysregulation of COL and, in particular, of CIV play an important role in this process [7,8,11,12,39].

We found in patients with T2D significantly lower levels of ACIVAbs IgM (Figure 1A) and CIV-DP (Figure 2A) compared to controls, which suggests an imbalance in the processes of synthesis and degradation of CIV in the vascular wall. We also found lower levels of ACIVAbs IgG compared to controls without being significant (Figure 1B). Identical to our results with lower but non-significant levels of ACIVAbs IgM and ACIVAbs IgA in patients with T2D than in controls have been also reported [33]. These data indicate that vascular metabolism of CIV is decreased in T2D with long-term complications, which probably leads to its excessive deposition in the BMs of the microcirculation. A similar hypothesis is further supported by the data on reduced serum CIC-CIV levels that we found in patients with T2D (Figure 2B). To support these conclusions, we examined this portion of ACIVAbs IgG that remain unbound with CIV-DP in the blood circulation and do not participate in the formation of CIC-CIV (free ACIVAbs IgG). The results showed that the levels of free ACIVAbs IgG were significantly higher compared to those of the healthy controls (Figure 2C). A possible explanation for these data is that as a result of reduced CIV degradation, most of the circulating ACIVAbs IgG remain unbound to metabolites of CIV, leading to their increased serum levels. Experimental data from tissue samples also support our results. It was found that BMs of retinal vessels in diabetic rats contain increased amounts of the α1 (IV) chain of collagen, as well as of the β1 and γ chains of laminin and fibronectin, indicating increased accumulation of matrix components [36,40]. It has also been reported that collagens III and IV in the kidneys of streptozotocin (STZ)-diabetic rats accumulate to a greater extent than in those of control rats [36,41]. In humans, it has been demonstrated that CIV along with collagens type I, III, and V was upregulated in the retinas of patients with DR compared to that in retinas of non-diabetic subjects [7,8]. In DN, podocytes adopt a “promatrix phenotype”, followed by preferential deposition of CIV in glomerular basement membrane (GBM) [20,30,42].

In diabetes, vascular BM thickening occurs not only due to excessive synthesis and deposition of ECM components, but also due to their decreased degradation [20,38]. Hyperglycemia-induced dysregulated of MMPs may be an important factor for increased CIV deposition in the vascular BMs [30]. The subgroup of MMPs known as gelatinases, in particular gelatinase A (MMP-2) and gelatinase B (MMP-9), can degrade COL and denatured COL (gelatin), and their altered activity might be implicated in the pathophysiology of diabetes complications [15]. In our study, we found significantly higher serum levels of MMP-2 and MMP-9 in the T2D patients compared to controls (Table 1) and a positive correlation between MMP-2 and ACIVAbs IgM in the T2D group, showing the linear relationship between MMP-2 levels and vascular CIV turnover (Figure 1C). A possible explanation for these results is that both gelatinases could be involved in the development of vascular complications in T2D, but with a predominant role of MMP-2 in CIV metabolism. Considering this correlation at the biological level, it can be concluded that the local regulation of MMP-2 activity may play an important role in the pathogenesis of diabetic vascular complications by affecting CIV metabolism in the vascular wall. In this regard, some authors suggest that MMP-2 may be a good index of the severity of microangiopathy, and MMP-9 may be a marker of macroangiopathy in diabetes [43]. In diabetes with long-term complications, increased expression of tissue inhibitors of MMPs (TIMPs) has been observed, which may suppress enhanced MMP activity [44]. Elevated circulating levels of MMP-2 and MMP-9 along with elevated levels of TIMP-1 [45,46,47] were observed in patients with DR compared to diabetic patients without retinopathy. Data suggesting a link between MMP-2 dysregulation and DN also exist. Rodent models of diabetes reveal decreased expression and/or proteolytic activity of MMP-2 in renal tissues [48], suggesting decreased degradation of CIV in the GBM. In patients with DN, increased expression and activity of TIMP-1, TIMP-2, and TIMP-3 can contribute to excessive deposition of ECM components in GBM [20]. Decreased activity of MMP-2 and MMP-9 is observed with increased activity of TIMP-1, which leads to excessive deposition of CIV and fibronectin in the GBM [49]. TIMP-2, which is a specific inhibitor of MMP-2, was strongly increased in glomerular epithelial cells cultured in high glucose and also in the kidneys of STZ-diabetic rats [36]. TIMP-3, the most highly expressed TIMP in the kidney, was upregulated in both experimental and human DN. In diabetic mouse models, TIMP-3 knockout results in GBM thickening and albuminuria [20]. Systemic concentrations of MMP-2 and MMP-9 were also increased in peripheral arterial disease and HTN, which are present in a significant proportion of patients with T2D [50,51]. In addition, glucose-induced modifications of the vascular ECM not only result in increased deposition of structural proteins, but also renders glycated components, such as CIV, less susceptible to proteolysis by MMPs [36].

The strength of our study is that it describes a specific immunological method for examining vascular CIV turnover in patients with T2D. A limitation of the study is the relatively small number of studied persons, which requires these results to be confirmed in larger studies.

## 5. Conclusions

Given the severe consequences of vascular damage in patients with T2D, new non-invasive methods are needed to assess vascular changes. The study of vascular metabolism of CIV by measuring the levels of specific immunological biomarkers such as ACIVAbs, CIV-DP, CIC-CIV, and free ACIVAbs can be used to monitor the development of vascular complications and the therapeutic response in patients with T2D. Our research of these biomarkers showed significantly lower serum levels of ACIVAbs IgM, CIV-DP, CIC-CIV, and significantly higher levels of free ACIVAbs IgG in the T2D group compared to controls. These results suggest that vascular metabolism of CIV is decreased in patients with long-term complications of T2D, which may lead to its excessive accumulation in capillary BMs and their thickening. The data also suggest that MMP-2 may be involved in the development of vascular complications in T2D, which is confirmed by the existence of a positive linear relationship between the levels of MMP-2 and ACIVAbs IgM. Considering this correlation at the biological level, it can be concluded that up- or downregulation of MMP-2 activity may play an important role in the pathogenesis of diabetic vascular complications by affecting CIV metabolism in the vascular wall.

## Figures and Tables

**Table 1:** Clinical characteristics of the groups in the study population.

Variables	Healthy Control Subjects	Patients with T2D
	(*n* = 20)	(*n* = 59)
Men, *n* (%)	10 (50.0)	25 (42.0)
Women, *n* (%)	10 (50.0)	34 (58.0)
Age, years ^1^	61.5 ± 11.4	60.8 ± 14.7
Duration of T2D ^1^	N/A ^2^	10.1 ± 7.8
SBP, mmHg ^1^	121.5 ± 8.6	149.2 ±16.7 ***
DBP, mmHg ^1^	78.2 ± 7.5	83.0 ± 10.4
BMI, kg/m^2 1^	24.9 ± 2.4	28.4 ± 4.5 ***
Smokers, *n* (%)	4 (20)	9 (15)
HbA1c (%) ^1^	N/A ^2^	7.5 ± 1.8
TC, mmol/L ^1^	4.2 ± 0.7	5.2 ± 1.8 *
LDL-C, mmol/L ^1^	2.8 ± 0.8	3.0 ± 1.1
HDL-C, mmol/L ^1^	1.2 ± 0.2	1.0 ± 0.3 ***
TG, mmol/L ^1^	1.4 ± 0.4	2.7 ± 3.0
CRP, mg/L ^1^	1.1 ± 0.9	8.4 ± 7.9 ***
MMP-2, ng/mL ^1^	30.68 ± 8.4	36.22 ± 11.5 *
MMP-9, ng/mL ^1^	25.84 ± 12.7	38.48 ± 20.7 **
Neuropathy, *n* (%)	N/A ^2^	8 (14.0)
Microangiopathy, *n* (%)	N/A ^2^	50 (85.0)
Macroangiopathy, *n* (%)	N/A ^2^	18 (31.0)

* *p* < 0.05, ** *p* < 0.01; *** *p* < 0.001; ^1^ Mean ± SD; ^2^ N/A, not available; SBP: systolic blood pressure; DBP: diastolic blood pressure; BMI: body mass index; TC: total cholesterol; LDL-C: low-density lipoprotein cholesterol; HDL-C: high-density lipoprotein cholesterol; TG: triglyceride; CRP: C-reactive protein; MMP-2: matrix metalloproteinase-2; MMP-9: matrix metalloproteinase-9.

## Data Availability

The authors confirm that the data supporting the findings of this report are available within the article.
